# Medical system and nutrition improvement for the rural elderly

**DOI:** 10.1186/s41043-019-0189-x

**Published:** 2019-10-18

**Authors:** Zhenhua Wang, Jinqi Jiang, Qiyan Zeng

**Affiliations:** 1 College of Economics and Management, Shenyang Agricultural University, Shenyang, 110866 China; 2 College of Economics and Management, Zhejiang A&F University, Hangzhou, 311300 China

**Keywords:** Aging, The new rural cooperative medical system, The rural elderly, Nutrition improvement

## Abstract

**Background:**

Insufficient nutrition intake has negatively influenced the health of the elderly in rural China where the problem of population aging is serious. The present study aims to explore whether the medical system, called the New Rural Cooperative Medical System (NRCMS), can improve the rural elderly’s nutrition intake and the mechanism behind it.

**Methods:**

The difference in differences (DID) model and the propensity score matching-difference in differences (PSM-DID) model are both performed to investigate the impact of the medical system on nutrition improvement for the rural elderly. Two thousand seven hundred eighty rural elderly samples tracked in 2000 and 2006 from the China Health and Nutrition Survey are analyzed. Indices for the elderly’s nutrition intake includes daily average intake of energy, fat, protein, and carbohydrate.

**Results:**

The results show that participation in the NRCMS can significantly increase the rural elderly’s total energy intake, carbohydrate intake, and protein intake by 206.688 kcal, 36.379 g, and 6.979 g, respectively. A more significant impact of the NRCMS on nutrition intake is observed in the central and near-western where economic development is lagging behind. Also, compared to people of 18–60 age group, such impact is statistically more significant in the elderly for the carbohydrate intake.

**Conclusions:**

The NRCMS can improve the rural elderly’s nutrition intake in China. As the population ages rapidly in rural China, the present study provides recommendations on how to improve nutrition and health status of the elderly from the aspect of the medical system.

## Background

China has the largest elderly population in the world. According to the data of National Bureau of Statistics, the national population aged 60 and over has reached 222 million in 2016, accounting for 16.15% of the total population. Also, population aging is more pronounced among the rural population than in the urban population [1]. The rural population aged 60 and over has accounted for 18.47% of the total rural population, which is 4.13% higher than that in the urban areas. Considering the aggravating aging problem and relatively backward development in rural China, how to guarantee the nutrition and health of huge rural elderly populations has become social focus.

Under the current social and economic development situation in rural China, the elderly population have unique characteristics. On the one hand, the elderly have low economic income and basically rely on children’s support [2]. The majority of the rural elderly also need to work on the farms to make a living. On the other hand, elderly empty nesters are easy to find in rural China. Massive numbers of rural youth have left home to work in the cities and leave behind their elder parents of whom no one takes care [3]. In addition, the rural elderly who have experienced the Great Chinese Famine and the subsequent resource shortages are often excessively thrifty and have very little knowledge about nutrition and health.^1^ These effects have contributed to the pessimistic nutrition outlook for China’s rural elderly, especially reflecting in insufficient nutrition intake [4–6]. The undernutrition rate of China’s rural elderly aged 60 and over was 6.4% in 2010, which was higher than that of their counterparts in the urban areas (3.3%) and higher than other age groups in rural China [6]. Undernutrition has posed serious health threats on the rural elderly population, such as reduced immune function, increased morbidity and mortality for some diseases (anemia, cancer, angiocardiopathy etc.), and cognitive decline [6, 7]. As a result, it has been a great healthcare concern and a huge economic burden to the healthcare system in China.

Therefore, it is imperative to improve the nutrition intake of China’s rural elderly. The importance of income in promoting nutrition intake has been confirmed in numerous studies [8, 9]. Also, it is found that future income and expenses uncertainty are important factors that can impact food consumption and nutrition intake [10, 11]. For example, Meng et al. (2009) put forward that because of China’s reform and opening policy, the uncertainties resulting from market reforms in employment, pension, and other areas may be the important factors for the worsen of residents’ nutrition [10]. Based on these studies, the present study focused on exploring the impact of the New Rural Cooperative Medical System (NRCMS) on the rural elderly population’s nutrition intake, for medical insurance is supposed to alleviated future income and expenses uncertainty [12].

The NRCMS is the most important medical system for China’s rural residents in coping with disease and related medical expenses. Since medical expenses often cause financial hardship for many rural families [13], the Chinese government launched the NRCMS in 2003 to address the problem. The NRCMS is defined as a mutual help and risk-pooling health protection system. There are three specific guidelines for the implementation of the NRCMS: (1) participation in the NRCMS is voluntary; (2) the NRCMS would focus on catastrophic illnesses, receiving funding from both the government (central and local) and individuals; (3) the NRCMS subscribers are coming from larger risk-sharing pools at the county level. There is a rapid increase in the NRCMS coverage since 2003 and it has reached 98.7% of China’s rural areas by the end of 2013. The NRCMS has received strong financial support from the central and local governments, and thus the government subsidies, illnesses coverage, reimbursement percentage, and others have increased significantly over time. At began, the central government provided an annual subsidy of 10 Yuan for each person who joined the NRCMS and local governments in total paid at least 10 Yuan per person to match the individual premium of around 10 Yuan. Rural residents participated in the NRCMS could get some reimbursement for outpatient and hospital charges and catastrophic illnesses charges. In 2017, the central government has raised the NRCMS subsidy to RMB 450 per capita per year, and the reimbursement percentage for some outpatient and hospital charges reach approximately 50% and 75%, respectively. The uncertainty of rural residents’ future medical expenses is therefore greatly alleviated which may, in turn, impact their current food consumption [12].

Some studies have already found that the NRCMS can improve adults’ nutrition intake [11, 12]. By comparing energy intake between household enrolled in the NRCMS and non-participants, Ma and Zhang (2011) have found the NRCMS increases household per capita calorie, protein, and fat intake [12]. However, in some studies, it is discussed medical insurance may have a negative impact on people’s nutrition intake and health due to the presence of an ex-ante moral hazard [14]. That is, due to decline in the cost of being sick after joining the NRCMS, residents’ incentives to prevent from becoming sick (such as healthy dietary habits and exercise) weaken significantly, which becomes counter-productive to improving the nutrition intake [15].

In conclusion, taking whole adults as an example, previous studies have investigated the impact of the NRCMS on nutrition intake. However, the NRCMS’ impact on the rural elders’ nutrition intake may be different from the other age groups due to poor health, low economic capabilities, and lack of daily care for the rural elderly (as shown in paragraph 2). Yet, no related analysis has been performed so far. Therefore, considering the serious aging problem in rural China and the poor nutrition status of the elderly, the present study adopts panel data for China’s rural elderly and combines the difference in differences (DID) and propensity score matching-difference in differences (PSM-DID) models to analyze whether the NRCMS can improve the rural elderly’s nutrition intake. We also investigate whether the impact of the NRCMS on nutrition intake differs in different regions and age groups.

The remaining parts of this paper are organized as follows. Section 2 shows the data and methods, and Sections 3 and 4 present the estimation results and discussion, respectively. Section 5 concludes the paper.

## Data and methods

### Data

The data for this study were drawn from the China Health and Nutrition Survey^2^ (CHNS), which is an ongoing open cohort, international collaborative project between the Carolina Population Center at the University of North Carolina at Chapel Hill and the National Institute for Nutrition and Health at the Chinese Center for Disease Control and Prevention. The survey began in 1989 and was conducted every 2–4 years. The first nine rounds of the data have been published (1989, 1991, 1993, 1997, 2000, 2004, 2006, 2009, and 2011). The CHNS includes eight or nine diverse provinces that vary substantially in geography, economic development, and health indicators (Guangxi, Guizhou, Heilongjiang, Henan, Hubei, Hunan, Jiangsu, Liaoning, Shandong), and an additional three municipalities (Beijing, Shanghai, Chongqing) also join since 2011. A multistage cluster random sampling method is used to derive the original sample in the survey design,^3^ and the related information is collected by questionnaire survey on the individual, household, and community levels. The CHNS contains key variables information we need such as the NRCMS participation and food intake, which fully supports our analysis.

The pilot program of the NRCMS started in 2003, hence, in order to better capture the implementation effect, the present study used data from the 2000 survey (before the NRCMS implementation) and from the 2006 survey (after the NRCMS implementation). We did not choose data from 2004 because few rural elderly people had participated in the NRCMS in the CHNS (accounting for less than 5%). In other words, there were not enough samples in the treatment group. In 2006, however, more than 30% of the rural elderly participated in the NRCMS in the CHNS, which provided a sufficient number of samples for the treatment effects analysis. Also, although there were more samples participated in the NRCMS in the 2009 and 2011 survey, there is a significant sample loss for long time intervals because the samples are partly traced in the CHNS. Therefore, considering the present study’s need and the panel data availability, we have kept 2780 rural elderly samples (aged 55 and over in 2000, aged 61 and over in 2006 correspondingly) who were tracked in 2000 and 2006.

### Statistical analysis

The NRCMS can be viewed as a policy test and a DID model is commonly adopted to analyze the policy effectiveness in numerous researches [16]. In the present study, the rural elderly joining in the NRCMS are identified as the treatment group (*n* = 858), while others who do not participate in the program are in the control group (*n* = 1922). We assumed dnrcms is a dummy variable for the treatment status (dnrcms = 0 for the control group, and dnrcms = 1 for the treatment group). The samples are investigated at two time points, one before intervention and on after intervention. We define dt as a dummy variable for the survey year (dt = 0 for the year 2000, before the NRCMS implementation, and dt = 1 for the year 2006, after the NRCMS implementation). Thus, the DID model can be expressed as

1 $$ {Y}_{it}={\beta}_0+{\beta}_1{\mathrm{dnrcms}}_{it}+{\beta}_2{dt}_{it}+{\beta}_3{\mathrm{dnrcms}}_{it}\ast {dt}_{it}+{\beta}_4{X}_{it}+{\varepsilon}_{it} $$

In Eq. (1), the subscript *i* represents the *i*th sample and the subscript *t* is the year. *Y* represents the rural elderly’s nutrition intake, including daily average energy intake (kcal/day), daily average carbohydrate, fat, and protein intake (g/day). They are calculated based on the detailed individual food intake information through in-person interviewer-administered 24 h recalls conducted by trained staff over 3 consecutive days, including 2 working days and 1 weekend day. *X* is a set of control variables according to previous studies, including demographic variables (age, gender, education, health), socio-economic variables (income, household size, other medical insurance except for NRCMS), lifestyle variables (smoke, drink, activity level), and provinces dummies [10–12, 17]. The detailed definitions of these variables are presented in Table 1.

**Table 1:** Definition and summary statistics of variables used in the analysis

Variable	Definition	Mean	SD
Energy	Average daily energy intake (kcal/day)	2052.907	708.801
Carbohydrates	Average daily carbohydrate intake (g/day)	306.261	118.706
Fat	Average daily fat intake (g/day)	63.062	37.365
Protein	Average daily protein intake (g/day)	58.775	23.954
dnrcms	The treatment dummy, dnrcms = 0 for the control group, and dnrcms = 1 for the treatment group	0.309	0.462
dt	The year dummy, dt = 0 for 2000, and dt = 1 for 2006	0.500	0.500
dnrcms*dt	The interaction term between dnrcms and dt	0.154	0.361
Age	In years	67.749	7.829
Gender	Male = 0, female = 1	0.525	0.499
Education	years of formal education	3.364	3.793
Health	Self-assessed health condition, a vector of dummy variable, poor = 1, base; fair = 2; good = 3; excellent = 4	2.286	0.754
lnIncome	Per capital annual household income, in logarithm	8.084	1.052
Hhsize	Number of people in the household	3.594	1.913
Smoke	Currently smoking, yes = 1, no = 0	0.290	0.454
Drink	Drinking alcohol over last year, yes = 1, no = 0	0.273	0.445
Activity	A vector of dummy variable, no working ability = 1, base; light level = 2; moderate to vigorous level = 3	2.439	0.556
Insurance	Own of other medical insurances, yes = 1, no = 0	0.102	0.303
Province dummies	A vector of dummy variable	/	/

For the rural elderly who participate in the NRCMS, the change in their nutrition intake before and after the NRCMS implementation can be expressed as

2 $$ {\varDelta Y}_{i1}={Y}_{i1}-{Y}_{i0}=\left({\beta}_0+{\beta}_1+{\beta}_2+{\beta}_3\right)-\left({\beta}_0+{\beta}_1\right)={\beta}_2+{\beta}_3 $$

which includes the impact of the NRCMS and other factors on the elderly’s nutrition intake. For the rural elderly who do not participate in the NRCMS, the change in the nutrition intake before and after the NRCMS implementation is

3 $$ {\varDelta Y}_{i0}={Y}_{i1}-{Y}_{i0}=\left({\beta}_0+{\beta}_2\right)-{\beta}_0={\beta}_2 $$

which primarily describes the impact of other factors on the elderly’s nutrition intake. Therefore, *ΔY*_*i*1_ − *ΔY*_*i*0_ is generated as follows:

4 $$ {\varDelta \varDelta Y}_i={\varDelta Y}_{i1}-{\varDelta Y}_{i0}=\left({\beta}_2+{\beta}_3\right)-{\beta}_2={\beta}_3 $$

which shows the NRCMS’ impact on the elderly’s nutrition intake. If *β*_3_ is positive, then the NRCMS improves the rural elderly’s nutrition intake. If *β*_3_ is negative, the NRCMS worsen the rural elderly’s nutrition intake.

However, the validity of DID rests on “parallel time drift”, where a change in the control group between post-treatment and pre-treatment periods is identical with such change in the treatment group when there is no treatment. But such assumption is somewhat difficult to satisfy in reality; thus, the present study also adopts the PSM-DID method to resolve this issue [18, 19]. The PSM-DID model can construct more comparable treatment and control groups by conditioning on the propensity scores in the initial time period. Specifically, we firstly run a logit regression to obtain the estimates of the propensity scores with the dependent variable being participation in the NRCMS, and the independent variables being demographic, socio-economic, and regional characters.^4^ Then, the samples in the treatment and control groups are matched according to the propensity score, and we estimate the difference in average nutrition intake changes of the two matched groups before and after invention to investigate the effect of the NRCMS.

### Variables description

Daily average nutrition intake of the rural elderly in 2000 and 2006 is shown in Table 2. There was an obvious decrease in nutrition intake from 2000 to 2006. The daily average energy intake in 2000 was 2125.610  kcal, mainly consist of 318.556 g carbohydrates, 65.108 g fat, and 60.436 g protein, while it decreased to 1972.707 kcal in 2006, mainly containing 292.699 g carbohydrates, 60.805 g fat, and 56.943 g protein. The decline in the rural elderly’s nutrition intake exists in both treatment and control groups, and the latter decreased more rapidly.

**Table 2:** Daily nutrition intake of the rural elderly in 2000 and 2006

Variables	Total samples		Treatment group		Control group	
	2000	2006	2000	2006	2000	2006
Energy	2125.610 (702.041)	1972.707 (707.864)	2249.525 (717.270)	2182.116 (798.325)	2068.642 (687.876)	1857.009 (623.594)
Carbohydrates	318.556 (113.262)	292.699 (123.067)	340.248 (115.374)	330.940 (143.961)	308.583 (10.927)	271.572 (104.068)
Fat	65.108 (38.449)	60.805 (36.013)	66.561 (34.258)	64.306 (37.568)	64.440 (40.231)	58.871 (34.999)
Protein	60.436 (24.225)	56.943 (23.526)	63.615 (25.892)	62.958 (27.117)	58.974 (23.288)	53.619 (20.561)

Notes: Standard deviation in parentheses; unites of measurement for energy, carbohydrates, fat and protein are kcal/day, g/day, g/day, and g/day, respectively

Daily average energy intakes of elderly males and females in rural China were 2158.867 kcal/day and 1808.744 kcal/day, respectively, which was lower than the recommended amount of caloric intake for elderly males and females with moderate-intensity activity (2350 kcal/day for elderly males and 1950 kcal/day for elderly females) according to “Chinese dietary reference intakes^5^ (hereinafter referred to as the reference).” It confirmed the inadequate energy intake problem in the rural elderly in China.

Also, energy is mainly obtained from carbohydrates, protein and fat, and the reference has set standards for protein intake amount per day and the proportion of energy from carbohydrates and fat. It should be noted that China’s rural elderly were facing the serious problem of insufficient protein intake, which has been emphasized in previous studies (4). Elderly males and females’ daily protein intake were 61.791 g/day and 52.672 g/day, respectively; however, the recommended amount were 65 g/day and 55 g/day, respectively. Therefore, an appropriate increase in protein was expected to promote the rural elderly’s health. In addition, the proportion of energy from carbohydrates and fat^6^ were 59.35% and 27.74%, respectively, which is within the reasonable range recommended by the reference (20–30% for fat; 50–65% for carbohydrates).

## Results

The DID model is adopted to examine the effect of the NRCMS on the rural elderly’s nutrition intake. As the results shown in the Table 3, the NRCMS participation significantly increases the rural elderly’s daily energy intake (*β*_3_ = 206.686, *P* < 0.01). In terms of the macronutrients, the NRCMS has significantly positive effect on their daily intakes of carbohydrates (*β*_3_ = 36.379, *P* < 0.01) and protein (*β*_3_ = 6.979, *P* < 0.01), while it has no effect on fat intake (*β*_3_ = 4.867, *P* > 0.1). Specifically, the NRCMS participation could increase the rural elderly’s daily average energy intake by 206.686 kcal, including an increase of 36.379 g carbohydrate and 4.867 g protein intake, respectively.

**Table 3:** The impact of the NRCMS on the rural elderly’s nutrition intake based on a DID model

Dependent variables	Independent variables	Coef	SE	*P* value	95% CI
Energy	dnrcms*dt	206.686	59.483	0.001	(90.029, 323.343)
	dnrcms	12.092	46.542	0.795	(− 79.185, 103.368)
	dt	− 51.274	34.246	0.135	(− 118.437, 15.889)
	Control variables	Yes	–	–	–
	*R* ^2^	0.281	–	–	–
Carbohydrates	dnrcms*dt	36.379	9.799	0.000	(17.162, 55.596)
	dnrcms	− 4.404	7.373	0.550	(− 18.863, 10.055)
	dt	− 2.116	5.702	0.711	(− 13.299, 9.067)
	Control variables	Yes	–	–	–
	*R* ^2^	0.302	–	–	–
Fat	dnrcms*dt	4.867	3.315	0.142	(−1.634, 11.367)
	dnrcms	1.927	2.440	0.430	(−2.858, 6.711)
	dt	− 5.004	2.055	0.015	(− 9.035, − 0.973)
	Control variables	Yes	–	–	–
	*R* ^2^	0.118	–	–	–
Protein	dnrcms*dt	6.979	2.182	0.001	(2.700, 11.258)
	dnrcms	− 0.553	1.764	0.754	(− 4.013, 2.907)
	dt	− 1.534	1.167	0.189	(− 3.823, 0.756)
	Control variables	Yes	–	–	–
	*R* ^2^	0.203	–	–	–
	Obs	1983			

Notes: Demographic variables, socio-economic variables, lifestyle variables and provinces dummies as shown in Table 1 are controlled in the DID model. *Coef* is abbreviated for coefficient

Considering the inadequate energy intake and undernutrition problem, participating in the NRCMS could play a positive role in improving the rural elderly’s nutrition status. Also, the increase in energy intake is achieved mainly by more carbohydrate and protein intake. Given the insufficiency in protein intake in the diet, an increase in protein intake after joining the NRCMS is especially expected to promote nutrition status.

The results of control variables are basically consistent with the existing researches [10–12, 17]. For instance, the dietary energy and nutrients intake decrease with age. The elderly males have more energy and nutrients intake than the elderly females. Increase in income and own of other medical insurance significantly contribute to more fat and protein intake, while poor health leads to less nutrients intake.

Furthermore, to construct more comparable treatment and control groups and lower the DID estimation bias, the present study also adopts the PSM-DID method for robustness check. During the experiment process, a logit estimation is performed on the DID model control variables to find out whether the NRCMS participation would generate a propensity score.^7^ The logit estimation results show that the covariates have strong explanatory powers on the treatment variables. In addition, after the test of propensity score matching, no significant difference in the covariates average value between the control and the treatment groups is discovered. This result indicates an equal variables distribution after matching between the treatment and the control groups, and the validity of the PSM-DID method is confirmed [18]. We adopt kernel matching for the estimation in this section.

The results of the PSM-DID estimation are shown in Table 4. The NRCMS participation significantly improves the rural elderly’s energy intake by 165.356 kcal. For food macronutrients, the NRCMS participation could increase the rural elderly’s daily average carbohydrates and protein intake by 29.772 g and 6.151 g, respectively, but no significant impact was found for fat intake. These results are consistent with the results of DID model, showing good robust of our analysis.

**Table 4:** The impact of the NRCMS on the rural elderly’s nutrition intake based on the PSM-DID model

Dependent variables	Outcome variables	Coef	SE	*P* value
Energy	Before: differ (treated-control)	21.562	66.141	0.744
	After: differ (treated-control)	186.918	71.438	0.009
	Diff-in-diff	165.356	97.355	0.090
Carbohydrates	Before: differ (treated-control)	− 1.030	11.245	0.927
	After: differ (treated-control)	28.742	13.599	0.035
	Diff-in-diff	29.772	17.646	0.092
Fat	Before: differ (treated-control)	2.259	2.974	0.448
	After: differ (treated-control)	5.796	3.689	0.116
	Diff-in-diff	3.537	4.738	0..455
Protein	Before: differ (treated-control)	− 1.684	2.607	0.518
	After: differ (treated-control)	4.468	2.476	0.071
	Diff-in-diff	6.151	3.595	0.087

Notes: *before* means before the implement of the NRCMS, and *after* means after the implement of the NRCMS

## Discussion

### The impact of the NRCMS on rural elderly’s nutrition intake

The empirical results have proven that the NRCMS can significantly increase rural elderly’s nutrition intake in China, which introduces a new way to improve China’s huge rural elderly population’s nutrition and health. The NRCMS can influence the rural elderly’s nutrition intake in many ways. First, the NRCMS can lower future medical expenses uncertainty. Before the implement of the NRCMS, the rural elderly had hardly any medical insurance and would rationally increase their savings as a “self-insurance” [20]. As a social security system, the NCRMS help reduce medical expenses incurred by rural households, which greatly reduces the elderly’s future medical expenses uncertainty [12]. As a result, the rural elderly would lower their preventive savings and increase current consumption such as food that accounts for the major portion of the rural elderly’s total consumption. Second, the rural elderly’s current medical expenses are reduced [11, 20]. Unlike other commercial insurance policies, the NRCMS provides participated rural residents with outpatient services and hospitalization at specified institutions that only charge the program participants the net amount after reimbursements according to the NRCMS plan. In other words, rural residents do not need to prepay the full amount up front and get reimbursed later, which directly reducing their current medical expenses. As a result, the elderly will have more money to spend on other types of consumption including food, thereby improving their nutrition intake. Third is the government subsidy’s income effect of the NRMCS, which is also known as the transfer payment effect [11, 12]. It is equivalent to an increase in income, which creates an income effect conducive to more food consumption and nutrition intake for the elderly. Fourth, the NRCMS participation improves the rural elderly’s nutrition knowledge and healthy dietary awareness that may promote their nutrition intake. The NCRMS provides free regular checkups and health consultation, which help the rural elderly get in contact with medical personnel. Therefore, it helps to increase the elderly’s health knowledge and enhance understanding of healthy diet, thereby enhancing their incentives to improve nutrition intake.

It should also be mentioned that the present study found no significant ex-ante moral hazard related to the NRCMS in the rural elderly samples in China. There are some possible reasons. First, the rural elderly are still experiencing the problem of inadequate nutrition intake [4, 6]. Therefore, the health risks resulting from excessive nutrition intake mentioned in the studies about the NRCMS do not happen at present. Second, the elderly’s physical functions are relatively weak and they are at high health risks resulting from smoking, drinking, and excessive fat intake [21]. As a result, the elderly are more motivated to protect themselves and prevent diseases.

### Comparison of different age groups

The present study further compares the nutritional impacts of the NRCMS on rural elderly with that on other age groups (aged 18–55 in 2000). As shown in Table 5, except for fat intake, it is obvious that the coefficient value for the NRCMS effect of the 18–60 age group is smaller than that of the rural elderly. The results based on the seemingly unrelated estimation (abbreviated as “suest”) further confirm the statistically significant difference between the two subgroups in the effect of the NRCMS on the carbohydrate intake (*P* < 0.1), but not energy, protein, and fat intake (*P* > 0.1). It indicates the NRCMS plays a more significant role in increasing the rural elderly’s carbohydrate intake (a main source of energy) than other age groups.

**Table 5:** The impact of the NRCMS on the nutrition intake for adults (aged between 18 and 55 in 2000) based on a DID model

Dependent variables	Independent variables	Coef	SE	*P* value	95% CI
Energy	dnrcms*dt	166.152	30.973	0.000	(105.436, 226.869)
	dnrcms	38.184	24.195	0.115	(− 9.246, 85.613)
	dt	− 135.492	20.189	0.000	(− 175.070, − 95.915)
	Control variables	Yes	–	–	–
	*R* ^2^	0.205	–	–	–
Carbohydrates	dnrcms*dt	15.436	5.270	0.003	(5.105, 25.767)
	dnrcms	9.828	4.117	0.017	(1.758, 17.899)
	dt	− 24.258	3.435	0.000	(− 30.992, − 17.524)
	Control variables	Yes	–	–	–
	*R* ^2^	0.230	–	–	–
Fat	dnrcms*dt	8.375	1.793	0.000	(4.859, 11.890)
	dnrcms	− 0.422	1.401	0.763	(− 3.169, 2.324)
	dt	− 3.766	1.169	0.001	(− 6.057, − 1.474)
	Control variables	Yes	–	–	–
	*R* ^2^	0.133	–	–	–
Protein	dnrcms*dt	5.582	1.050	0.000	(3.523, 7.640)
	dnrcms	0.0003	0.820	1.000	(− 1.608, 1.608)
	dt	− 2.384	0.684	0.000	(− 3.725, − 1.042)
	Control variables	Yes	–	–	–
	*R* ^2^	0.173	–	–	–
	Obs	6633			

Notes: Demographic variables, socio-economic variables, lifestyle variables, and provinces dummies as shown in the Table 1 are controlled in the DID model

We provide possible explanations in the following two aspects according to the mechanism discussed above and the rural elderly’s characteristics. First, the elderly are more likely to get sick and future medical expenses uncertainty is higher for the elderly compared to any other age groups. At the same time, the rural elderly’s bodily function is weakening and they are less economically capable. Therefore, they are less resilient in fighting against uncertainties [22, 23]. The literature shows that an institutional arrangement lowering future uncertainties has a greater impact on lower income groups who are more vulnerable to future uncertainties [24]. Therefore, combined with the rural elderly’ economic status, the impact of the NRCMS on carbohydrate intake, a relatively cheap source of energy, is stronger among China’s rural elderly than other age groups. Second, according to the consumption theory, increase in income among different income level groups leads to various patterns of consumption [12]. The premium subsidy provided by the NRCMS is a type of transfer payment, and the resulting income effect is different among rural residents who participated in the NRCMS. Compared to other groups, the elderly’s income is relatively low and therefore, the NRCMS premium subsidy income effect is stronger for the elderly group compared to younger age groups.

### Comparison of different regions

Due to China’s vast territory and regional economic development policy in history, economic and social development levels are different across different areas. The NRCMS implementation also varies, for local governments are charged with setting up their own policy implementation plans. Therefore, the present study further examines if there are regional differences in the effect of the NRCMS on the rural elderly’s nutrition intake. Specifically, we divide China into the northeastern and eastern coastal region, and the central and near-western region according to the “new three regions” division proposal [25]. In 2006, the NRCMS participation rate of the rural elderly in the northeastern and eastern coastal region was 42.57%, while it was only 24.47% in the central and near-western region. Besides, the elderly’s daily average energy intake in the northeastern and eastern coastal region was 2082.084 kcal/day, which was significantly higher than that in the central and near-western region (2036.77 kcal/day, *P* < 0.01). As the regression results are shown in Table 6, the NRCMS does not have any significant impact on the rural elderly’s nutrition intake in the northeastern and eastern coastal region, but it has a significant positive impact on the elderly in the central and near-western region. The “suest” tests confirm the statistically significant difference between the two subgroups in the effect of the NRCMS on the energy intake (*P* < 0.01), carbohydrate (*P* < 0.01), fat (*P* < 0.1), and protein (*P* < 0.01). A possible reason could be that the rural elderly’s energy intake has basically satisfied their bodies’ needs and the Engel’s coefficient is relatively low in the northeastern and eastern coastal region where the economy is relatively developed. Thus, when uncertainty about the future is reduced, the rural elderly would choose to consume more durable goods or entertainment rather than food [26]. As a result, the NRCMS participation has little significant impact on total energy intake. However, the rural elderly’s energy intake level is relatively low in the central and near-western region that is less developed, and when uncertainty about the future is reduced, the rural elderly prefer to increase their energy intake to meet their bodies’ needs by consuming more food. In addition, the transfer payment effect of the NRCMS is stronger in the central and near-western region, which especially reflects in more financial subsidies from the central governments [27].

**Table 6:** Comparison of the NRCMS’ impact on the rural elderly’s nutrition intake across different regions

Dependent variables	Independent variables	Northeastern and eastern coastal region				Central and near-western region			
		Coef	SE	*P* value	95% CI	Coef	SE	*P* value	95% CI
Energy	dnrcms*dt	77.764	94.923	0.413	(− 108.608, 264.136)	267.383	80.113	0.001	(110.213, 424.553)
	dnrcms	36.385	75.518	0.630	(− 111.887, 184.657)	8.944	59.433	0.880	(− 107.655, 125.543)
	dt	40.266	68.433	0.556	(− 94.096, 174.628)	− 84.788	40.215	0.035	(− 163.685, − 5.892)
	Control var	Yes	–	–	–	Yes	–	–	–
	*R* ^2^	0.332	–	–	–		0.257		
Carbohydrates	dnrcms*dt	25.558	16.412	0.120	(− 6.667, 57.782)	31.029	12.410	0.013	(6.681, 55.376)
	dnrcms	− 9.966	12.395	0.442	(− 34.303, 14.372)	5.831	8.998	0.517	(− 11.821, 23.483)
	dt	15.847	12.084	0.190	(− 7.879, 39.584)	− 7.468	6.588	0.257	(− 20.392, 5.356)
	Control var	Yes	–	–	–	Yes	–	–	–
	*R* ^2^	0.296	–	–	–	0.326			
Fat	dnrcms*dt	0.381	4.985	0.939	(− 9.406, 10.169)	11.635	4.700	0.013	(2.414, 20.856)
	dnrcms	7.794	3.791	0.040	(0.351, 15.238)	− 3.239	3.211	0.313	(− 9.539, 3.061)
	dt	− 7.029	3.796	0.064	(− 14.482, 0.423)	− 5.140	2.434	0.035	(− 9.915, − 0.365)
	Control var	Yes	–	–	–	Yes	–	–	–
	*R* ^2^	0.200	–	–	–	0.097			
Protein	dnrcms*dt	4.609	3.399	0.175	(− 2.064, 11.282)	7.222	2.971	0.015	(1.393, 13.051)
	dnrcms	− 1.822	2.829	0.520	(− 7.376, 3.732)	0.456	2.282	0.842	(− 4.021, 4.932)
	dt	1.245	2.360	0.598	(− 3.388, 5.878)	− 2.420	1.354	0.074	(− 5.077, 0.237)
	Control var	Yes	–	–	–	Yes	–	–	–
	*R* ^2^	0.239	–	–	–	0.182	–	–	–
	Obs	709				1274			

Notes: Demographic variables, socio-economic variables, lifestyle variables, and provinces dummies as shown in the Table 1 are controlled in the DID model

### Limitations

This study suffers from several limitations: first, to estimate the causal effect of the NRCMS on the rural elderly’s nutrition intake, we mainly use the DID model. However, this is an observational study, not a randomized comparison, if self-selection that the elderly with more nutrition intake are more likely join the NRCMS is at work, a simple comparison between the characteristics of participants and non-participants cannot reveal any causal impact of the NRCMS on nutrition intake. Given the limitation, a credible test of the post-entry explanation should try to take into account possible biases stemming from self-selection. In order to reduce this bias (that is due to the observational nature of this study), we also combine the “selection on observables” with the “selection on time constant unobservables” hypotheses by employing the PSM-DID estimator introduced by Heckman et al. (1997). In other words, we will assume that conditional on observables the bias stemming from unobservables is the same in different time periods before and after the decision to export [28]. In this way, we can better evaluate the impact of the NRCMS on the rural elderly’s nutrition intake in face of possible residual confounding problems.

Second, the representative of data on food consumption can be a concern. The mean of 3-day food consumption data based on 24-h recall is used to proxy each person’s daily diet here, which may be prone to a misclassification errors due to seasonal variation in food consumption. Fortunately, the CHNS data are collected in autumn, a period in which food availability differences are minimized [29], and the mean of the intake distribution drawn from a large, representative sample of a group is not affected by day-to-day variation [30]. Also, the CHNS have collected food intake information for both working day and weekend day through in-person interviewer-administered 24-h recalls conducted by trained staff, which can enhance representation of food intake in a week. Besides, the measurement error caused by discrepancies between the self-reported dietary recall and the actual food consumption may differ among people of different characteristics and personalities, thus causing bias in our results. It widely exists in numerous researches related nutrition intake, and the large number of sample and professional survey methods in the CHNS are expected to ease the problem [31, 32].

In addition, due to some irresistible reasons (such as death and moving away,), about 30% of the elderly samples who were in the 2000 survey were not traced in the 2006 survey. We just keep the samples traced in 2000 and 2006, which may introduce possible sample selection bias. We try to delete samples as little as possible to ease the problem and such data processing is common in numerous studies based on the CHNS [33, 34].

Despite the limitation, our findings can still be suggestive by firstly investigating the relationship between the medical system and the elderly’s nutrition intake in terms of implications about the nutrition improvement for the rural elderly.

## Conclusion

China’s rural population is aging rapidly. Compared to younger groups, China’s rural elderly are facing specific problems of inadequate energy intake and undernutrition that have negatively affected their health and brought about serious social and economic burdens. The present study has confirmed that the NRCMS can significantly increase the rural elderly’s total energy, carbohydrate, and protein intake. Serving as a social security system for rural residents that helps the rural elderly cope with unexpected and huge medical expenses, the NRCMS can, to a certain extent, alleviate the rural residents’ future uncertainties and thereby improve their current consumptions including food consumption. Meanwhile, the present study’s findings also show that the NRCMS’ impact on the carbohydrate intake of rural elderly is stronger than people of 18–60 age group. In addition, such impact varies among the different regions; a more significant impact is observed in the central and near-western where economic development is lagging behind.

The rural elderly’s nutrition intake status is still not optimistic in China. Based on the present study’s findings, the following suggestions are put forward: (1) it is important to improve the NRCMS to increase medical security for the elderly and introduce more institutional arrangements specifically to lower the elderly’s risk of uncertainty such as insurance for major illnesses and accidental injuries; (2) when improving the NRCMS, more attention should be devoted to promote nutrition and health knowledge in order to maximize the NRCMS’ positive role in improving the rural residents’ nutrition intake; (3) the central government should increase the NRCMS subsidy amount for those rural residents living in regions with lower level of economic development; governments in less developed regions should also introduce more measures to promote the NCRMS and other systems implementation.

## Data Availability

The datasets analyzed during the current study are open access and available on the following website: http://www.cpc.unc.edu/projects/china/accessibility-info.

## Abbreviations

CHNS: The China Health and Nutrition Survey
DID: Difference in differences model
NRCMS: The New Rural Cooperative Medical System
PSM-DID: The propensity score matching-difference in differences model

## Footnotes

1 Based on “China’s 2014 Rural Endowment Status Report” released in the “Thousand Villages Investigation” conducted by the Shanghai University of Finance and Economics.

2 For further details on the CHNS, please visit its official website at http://www.cpc.unc.edu/projects/china.

3 A multistage, random cluster process is used to draw the original samples surveyed. Countries in each province are stratified by income (low, middle, and high), and a weighted sampling scheme is used to randomly select four counties. In addition, the capital and a lower income city of a province are selected when feasible, except that other large cities rather than capitals have to be selected in two provinces. Four villages and townships within the counties and urban/suburban neighborhoods within the cities are selected randomly. Twenty households are selected randomly in each candidate community and all household members are interviewed. More details of the survey design are available on the official website of the CHNS.

4 Demographic, socio-economic, and regional characters here include age, gender, education years, health status, income, household size, physical activity, provinces dummies, etc.

5 It is issued by National Health Commission of the People’s Republic of China. Chinese dietary reference intakes set standards for energy and protein amount per day, the proportion of carbohydrates and fat.

6 Energy is mainly got from three nutrients: carbohydrates, protein, fat, and the converted coefficients are 4 kcal/g, 4 kcal/g, 9 kcal/g, respectively. Energy from each nutrition divided by the total energy intake is the corresponding nutrition ratios.

7 Due to length limit, specific estimation results were not shown. Interested readers can contact the author for details.
